# Chemogenetic modulation of sensory afferents induces locomotor changes and plasticity after spinal cord injury

**DOI:** 10.3389/fnmol.2022.872634

**Published:** 2022-08-26

**Authors:** Jaclyn T. Eisdorfer, Hannah Sobotka-Briner, Susan Schramfield, George Moukarzel, Jie Chen, Thomas J. Campion, Rupert Smit, Bradley C. Rauscher, Michel A. Lemay, George M. Smith, Andrew J. Spence

**Affiliations:** ^1^Department of Bioengineering, College of Engineering, Temple University, Philadelphia, PA, United States; ^2^Department of Cell Biology and Neuroscience, Rutgers University, Piscataway, NJ, United States; ^3^Department of Neuroscience, Shriners Hospitals Pediatric Research Center, Lewis Katz School of Medicine, Temple University, Philadelphia, PA, United States

**Keywords:** spinal cord injury, clozapine-*N*-oxide, DREADDs or chemogenetics, Designer Receptors Exclusively Activated by Designer Drugs, kinematics, functional recovery after SCI, plasticity, sensorimotor

## Abstract

Neuromodulatory therapies for spinal cord injury (SCI) such as electrical epidural stimulation (EES) are increasingly effective at improving patient outcomes. These improvements are thought to be due, at least in part, to plasticity in neuronal circuits. Precisely which circuits are influenced and which afferent classes are most effective in stimulating change remain important open questions. Genetic tools, such as Designer Receptors Exclusively Activated by Designer Drugs (DREADDs), support targeted and reversible neuromodulation as well as histological characterization of manipulated neurons. We therefore transduced and activated lumbar large diameter peripheral afferents with excitatory (hM3Dq) DREADDs, in a manner analogous to EES, in a rat hemisection model, to begin to trace plasticity and observe concomitant locomotor changes. Chronic DREADDs activation, coupled with thrice weekly treadmill training, was observed to increase afferent fluorescent labeling within motor pools and Clarke's column when compared to control animals. This plasticity may underlie kinematic differences that we observed across stages of recovery, including an increased and less variable hindquarters height in DREADDs animals, shorter step durations, a more flexed ankle joint early in recovery, a less variable ankle joint angle in swing phase, but a more variable hip joint angle. Withdrawal of DREADDs agonist, clozapine-*N*-oxide (CNO) left these kinematic differences largely unaffected; suggesting that DREADDs activation is not necessary for them later in recovery. However, we observed an intermittent “buckling” phenomenon in DREADDs animals without CNO activation, that did not occur with CNO re-administration. Future studies could use more refined genetic targeted of specific afferent classes, and utilize muscle recordings to find where afferent modulation is most influential in altering motor output.

## Introduction

Spinal cord injury (SCI) is often a permanent debilitating disorder that disrupts sensory and motor function below the level of injury. Damage to sensorimotor pathways after SCI causes changes in neural circuitry, acutely and chronically. Behavioral improvements are often marked by the strengthening and rewiring of damaged and spared connections (Waters et al., 1996; Burns et al., 1997; Bilchak et al., 2021). Recent advances in treatments for SCI have demonstrated potential for promoting recovery after injury. In particular, epidural electrical stimulation (EES) applied to the lumbosacral spinal cord mediates functional improvements in patients with chronic SCI (Herman et al., 2002; Courtine et al., 2009; Harkema et al., 2011; Karimi et al., 2013; Angeli et al., 2014; Crosbie et al., 2014; Possover, 2014; Grahn et al., 2017; Formento et al., 2018; Gill et al., 2018). EES works by recruiting large diameter peripheral afferents, such as group I and II proprioceptive afferents and group II cutaneous afferents, that enter through the dorsal roots (Bouyer and Rossignol, 1998; Rossignol et al., 2006; Capogrosso et al., 2016). Activation of these afferents is thought to modulate synaptic connections that ultimately drive agonist/antagonist muscle recruitment. Precisely which pathways are responsible is an open question; it may be relatively localized to the spinal cord, or it may be indirect; relying on sensory information sent to supraspinal centers for motor correction and learning (Eisdorfer et al., 2020). Thus, mapping the circuitry that EES influences is a critical goal for the field (Spataro et al., 2005; Thelin et al., 2011; Côt et al., 2017), with clinical import.

While the effects of electrical stimulation can be inferred with histological methods, it is difficult to know precisely which neurons have been stimulated. This makes pinning down pathways of plasticity harder. Genetically encoded tools make this possible: by fusing the tool with a fluorescent protein, neurons that are manipulated are also labeled (Haery et al., 2019). This motivated us to use the genetically encoded excitatory DREADDs receptor hM3Dq in an EES-like context. DREADDs (Designer Receptors Exclusively Activated by Designer Drugs) are engineered “chemogenetic” receptors that enable targeted neural modulation in freely behaving animals through selective binding of a ligand, in this case clozapine-*N*-oxide (CNO) (Roth, 2016). DREADDs expression was driven by adeno-associated virus (AAV), and due to the coupled fluorescent protein, facilitate characterization of targeted neurons and the second-order neurons they influence (Wu et al., 2020).

The aims of this study were 2-fold: (1) to determine whether activation of large diameter peripheral afferents with hM3Dq DREADDs influences recovery from a hemisection spinal cord injury in the rat model and (2) to begin to uncover any underlying mechanisms of plasticity. Connections between afferents and motor pools or interneuronal networks may have important implications for functional recovery after injury. In particular, afferent activation impinging upon motor pools, either monosynaptically or polysynaptically, could increase appropriate activation/inactivation of agonist/antagonist muscle groups during training and rehabilitation (Hultborn et al., 1971; Mears and Frank, 1997; Dimitrijevic et al., 1998; Guertin, 2012). Furthermore, afferent information relayed to supraspinal centers may contribute to increased coordination of movement, motor learning, and motor correction after injury (Brownstone et al., 2015; Bui et al., 2016; Fink and Cafferty, 2016; Côt et al., 2017; Kim et al., 2017; Asboth et al., 2018; Eisdorfer et al., 2020; Gao et al., 2021). For example, interneuronal networks within Clarke's column (nucleus dorsalis or Clarke's nucleus), located within the thoracic and lumbar spinal segments, contain dorsal spinalocerebellar (dSC) tract neurons that can relay proprioceptive sensory signaling from the hindlimb to cortical motor centers (Kim et al., 1986; Aoyama et al., 1988; Edgley and Gallimore, 1988; Bosco et al., 2000; Bosco and Poppele, 2003; Hantman and Jessell, 2010; Sengul and Watson, 2012). As such, sprouting and synaptogenesis within lamina that contain motor pools and Clarke's column could support recovery after SCI.

To achieve these aims, we expressed hM3Dq DREADDs in large diameter peripheral afferents innervating the lumbar spinal cord to gain an understanding of the influence of selective continuous activation of large-diameter afferents can have on the hindlimb after SCI. In animals expressing DREADDs, we report higher densities of fluorescent axons in the motor pools and Clarke's column of the lumbar spinal cord, which may indicate that increased activation of afferents by DREADDs resulted in increased afferent sprouting and synaptogenesis onto interneurons and motorneurons. Analyses of kinematics for five points on the hindlimb—anterior superior iliac spine (ASIS), greater trochanter (hip), knee, ankle, and metatarsophalangeal (MTP) joints—revealed that chronic DREADDs activation leads to increased height of the hindquarters (e.g., indicated by the ASIS and hip heights), putatively indicating increased motorneuron activation and muscle recruitment. DREADDs animals also display ankle joint angles that are closer to the pre-injury condition. This may suggest that increased afferent activation helps to promote appropriate ankle movements during the step cycle. Interestingly, animals with DREADDs exhibit a buckling phenomenon, or a collapse of the hindquarters, in the absence of DREADDs activation (e.g., by withholding CNO administration) as observed in a larger range of ASIS heights during treadmill locomotion. Future work, such as using cFOS to examine and map changes in interneuronal networks, could seek to more directly tie changes in kinematics to observed changes in plasticity. Furthermore, these data that activate both proprioceptive and exteroceptive afferents form a baseline data set against which further work that restricts expression to proprioceptive or other subsets of afferents can be compared.

## Materials and methods

### Subjects

Twenty male and female Long-Evans rats (200–225 g) were obtained from Charles River Laboratories Inc. (Wilmington, Massachusetts) and housed in pairs with access to food and water *ad libitum*. Animal holding rooms are maintained on a 12-h light/dark cycle and experiments were conducted during the light phase. Experiments and animal handling under experimental protocol #4675 (Dr. Andrew J. Spence) was in strict accordance of guidelines set by Temple University's Institutional Animal Care and Use Committee (IACUC) and National Institute of Health (NIH). Animals were immediately terminated if they reached predetermined humane endpoints.

### Tattoo and marker application

To identify hindlimb joints in kinematics recordings, we used markers as joint indicators. Markers were applied to the following joints: the iliac crest (ASIS), the greater trochanter (hip rotation center), the knee, the ankle, and the metatarsophalangeal joint (MTP or toe joint). Markers, and therefore hindlimb joints, were easily detected with computer software from surrounding skin pixels in the camera capture volume. We used a combination of tattoo application and Sharpie markings for marker application. Unlike sticky retroreflective markers, tattoos and Sharpie markings are advantageous as they do not agitate the animals and do not affect their locomotion. Tattoos are also advantageous as they are long-lasting.

Tattoos were applied using the General Rodent Tattoo System (Cat. No. ATS-3, Animal Identification and Marking Systems, Inc., Hornell, NY) with methods described by the manufacturer. In brief, animals were induced with 3.5–4.5% isoflurane anesthesia with oxygen flow at 1 l/min and maintained at 1.5–2%. The right hindlimb and right-side abdomen were shaved and the skin cleaned using a cleanser provided by the manufacturer (Animal Tissue Cleanser Concentrate, Animal Identification and Marking Systems, Inc., Hornell, NY) and pat dry with sterile gauze. The needle of the tattoo machine was generously coated with blue ink and the distal third of the needle was inserted perpendicular to the skin. The skin covering the iliac crest, greater trochanter, and knee joints were tattooed in 2 cm horizontal or vertical movements and dipped back into the ink as needed. Tattoos were not applied to the ankle and MTP as these joints can be marked with Sharpie with light restraint on the day of video capture. A liberal amount of triple antibiotic ointment was applied onto tattooed skin to prevent irritation and scab formation. Sharpie markings were applied over the tattoos and on the ankle and MTP joints immediately prior to video capture to ensure visualization in the camera capture volume during subsequent pose estimation and 3D reconstruction.

### Surgical procedures

Surgeries were performed under aseptic conditions. Animals were anesthetized with a combination of ketamine (100 mg/mL, Zetamine, Vet One, Boise, ID), xylazine (100 mg/mL, AnaSed, Lloyd Laboratories, Shenandoah, IA), and sterile saline *via* IP injection and maintained at this surgical level with supplemental doses as needed. Musculature and skin were closed with 4-0 chromic gut sutures (DemeTECH, Miami Lakes, FL) and surgical skin staples, respectively. Postoperatively, animals were administered 10 cc sterile saline, antibiotic (0.5 g Cefazonlin powder reconstituted in sterile saline, Cat. No. NDC #0143-9923-90, Hikma Pharmaceutical USA, Inc., Eatontown, NJ), and analgesic (Rimadyl, 1 mg tablet, Cat. No. MD150-2, Bio-Serv, Flemington, NJ).

#### DRG injection surgeries

We chose lumbar dorsal root ganglia (DRG) L2-L5 as candidates for hM3Dq DREADDs expression as these DRG innervate muscles relatively broadly across the leg (Lavrov et al., 2008; Nakajima et al., 2008; Courtine et al., 2009). Further, we chose adeno-associated virus serotype 2 (AAV2) as a transduction method for delivery of hM3Dq DREADDs into the DRG because this virus is reported to primarily target large diameter afferents when directly injected into the DRG (Akache et al., 2006; Jacques et al., 2012). Furthermore, in a prior publication (Eisdorfer et al., 2021), we show that this method does not transduce thermal nociceptive afferents as verified by the Hargreaves assay. Excitation of large diameter afferents in the lumbar DRG is proposed to underlie enhanced recovery with EES after spinal cord injury (Bouyer and Rossignol, 1998; Rossignol et al., 2006; Capogrosso et al., 2016). The viral constructs utilized the human synapsin (hSyn) promotor and a fluorescent reporter protein (mCherry) for immunohistological characterization. A skin incision of ~5 cm in length was made along the dorsal midline beginning from the first lumbar segment (L1). We gently incised the superficial muscular fascia and separated the paraspinal muscles to expose the lateral surface of the right L2 to L5 vertebrae and the dorsal surface of the medial portion of the transverse processes. Accessory processes descending from the L2-L5 vertebrae were removed with a 1 mm rongeur (Friedman bone rongeurs, Fine Science Tools). Using the same rongeurs, laminar bone was removed to expose the distal third of the DRG. Using 0.1 mm ultra-fine clipper scissors, we removed the fascia covering the DRG (Fine Science Tools, catalog number: 15300-00). Animals were then attached to stereotactic spinal clamps for DRG injections. With methods adapted in part from Gompf et al. (2015), co-injections of pAAV-hSyn-DIO-hM3D(Gq)-mCherry (Addgene plasmid #44361; Roth, 2016), scAAV-Cre [generously gifted to us by the Hu Lab (Miao et al., 2016)], and Fast Green FCF (#F7258, Sigma-Aldrich) were administered to the four right L2-L5 DRG. DRG were injected with hM3Dq DREADDs (excitatory, *n* = 8) or control AAV virus (pAAV-hSyn-mCherry, *n* = 6) using a micromanipulator. Each DRG was injected with 1 uL of solution at a flow rate of 20 nL/s. To allow for distribution of fluid and equalization of tissue pressure, the pipette tip was left in place for 5 min following injection.

To confirm that our viral approach is targeting medium to large diameter afferents that are mostly proprioceptive and exteroceptive sensory afferents, we colabeled injected DRGs with CGRP and Parvalbumin (PV), and carried out a Hargreaves thermal nociception assay (Supplementary Figure S1). The proportion of mCherry positive cell bodies (virally transduced) that were also CGRP positive was 6.6 ± 4.9% (mean ± SD; *N* = 10 DRGs from five rats, range one to three DRGs per rat). The proportion of mCherry positive cell bodies that were also PV positive was 59.1 ± 16.6% of cells (mean ± SD; *N* = 10 DRGs from five rats, two DRGs per rat). Finally, we found that activation of our DREADDs with injection of CNO (4 mg/kg) did not significantly decrease the paw withdrawal time in the Hargreaves thermal nociception assay [one way repeated measures ANOVA; *p* = 0.42; *F*_(2, 10)_ = 0.95; *N* = 6 rats; adapted from Eisdorfer et al., 2021, with detailed methods therein].

To verify that CNO is activating afferents in these DRGs we stained for cFOS in the spinal cord segments that are innervated by these DRGs and counted the cFOS+ cells, finding more cFOS+ cells in animals with excitatory DREADDs given CNO than in control animals with either injections into DRGs of control constructs without DREADDs (mCherry), or in naïve animals that did not have DRG injections (Supplementary Figure S2; a separate cohort of *n* = 2 animals per group; one-sided unpaired *t*-test; *t* = 3.6, *p* = 0.039; Antibodies Inc., # N486/32).

#### Hemisection SCI

A skin incision was made between spinal cord segments T4 – T12 and paravertebral muscles were retracted using 1 mm rongeurs (Friedman bone rongeurs, Fine Science Tools, Foster City, CA) to expose the dorsal side of the vertebral columns. A partial laminectomy was performed using the same rongeurs to expose spinal cord segments T9 – T11. Lidocaine was applied to the exposed spinal cord and followed by a complete hemisection of the right hemicord at T10 with a 25 Gauge 1.5” needle and Vannas Spring scissors (Cat. No. 15000-00, Fine Science Tools, Foster City, CA). Care was taken to ensure all appropriate tissue was cut. Recovery gelled food (DietGel, ClearH_2_O, Portland, ME) was provided to animals for 1 week following surgery. Bladder function is not compromised with this injury (Arvanian et al., 2009). We palpated the bladder and squeezed to ensure proper bladder function. We observed that the bladder was empty even from the beginning of injury. Chlorhexidine gluconate 0.2% (Dermachlor Rinse, Cat. No. 006356, Covetrus, Portland, ME) and liquid bandage (New-Skin, Cedar Knolls, NJ) was applied to the left hind paw throughout the course of the study to discourage autophagia. Animals were immediately terminated if they reach defined humane endpoints, such as the presentation of neurological signs of pain.

### Exercise training

Treadmill training consists of tri-weekly locomotion at the following 5 speeds: 16, 20, 24, 28, and 32 cm/s. Training sessions took ~25 min, with animals locomoting at each speed for 4 min, interleaved with 1 min recovery periods between speeds. Animals received an intraperitoneal injection (IP) injection of clozapine-*N*-oxide (CNO) at a dosage of 4 mg/kg 30 min prior to each of the 3 weekly sessions (MacLaren et al., 2016; Jendryka et al., 2019). Training took place in a multi-lane treadmill with individual lanes that are separated by plexiglass. Training concluded after 6 weeks post-injury.

### Behavioral outcomes

#### Kinematics recordings

3D kinematic data were captured on a custom color two-camera acquisition system at a frame rate of 250 Hz (Robertson, 2016). At the start of each day of motion capture, we calibrated the capture volume for subsequent 3D reconstruction of locations of features with sub millimeter accuracy (Hedrick, 2008). Movements were tracked in real-time using computer vision tools and observation to prevent animals from leaning on the plexiglass during walking bouts (Spence et al., 2013). Locomotion was captured synchronously by the two-camera high-speed system when an animal remained in the center of the treadmill belt for at least 4 s while the belt was in motion. Consistent, rapid, and objective videos of steady state locomotion are acquired for 5 trials at each of the 5 speeds.

#### Pose estimation

With DeepLabCut (DLC) methods described in Mathis et al. (2018) and Nath et al. (2019), we estimated the locations of the ASIS (iliac crest), hip (greater trochanter), knee, ankle, and MTP joints in kinematics recordings. In brief, the hindlimb joints were manually tracked in ~7200 frames with an image size of 2048 by 700 px [95% was used to train the ResNet-50-based model (He et al., 2015; Insafutdinov et al., 2016)]. A p-cutoff of 0.9 was used to gauge the effectiveness of joint estimation.

#### 3D reconstruction and kinematic analyses

With methods described by Maghsoudi et al. (2019), estimated 2D joint positions from each camera view and associated DLT matrices that calibrated each camera [with DLTcal in Matlab; (Hedrick, 2008)] were used to generate 3D reconstructions of joint locations. To cut the data intro strides, we used the conventional definition of a stride as a full cycle of one hindlimb movement, comprised of swing and stance phases (Hamers et al., 2006). The swing phase consisted of the time between when the right hindpaw is lifted off of the treadmill belt (toe off) to the time it contacted the belt again (toe on). The stance phase was the time between the initial contact of the right hindpaw with the belt to the time it lifted off again. The following features were computed with methods by Maghsoudi et al. (2019): angles for the ankle, hip, and ASIS; distances between adjacent joints; and the vertical distance between each of the joints and the treadmill belt (joint heights). Feature values are also calculated individually for swing and stance phases.

#### Necessity of DREADDs activation in late recovery

To determine whether CNO activation of DREADDs is required to for locomotor changes in later weeks, we ran the animals on the treadmill in the absence of CNO (−CNO) in the week following completion of exercise training (week 7). In week 8, we resumed administration of CNO (+CNO) and ran the animals on the treadmill again for kinematic data capture. Weeks 6, 7, and 8 therefore formed an ABA design withdrawal study, where the ABA design it used to mitigate history effects. To avoid progressing recovery due to continued treadmill training, we did not exercise animals in weeks 7 and 8, except for the 2 running bouts required to record kinematic data: without CNO (−CNO, week 7) and CNO administration (+CNO, week 8). In **Figures 3**–**5** which present kinematics, 7 and 8 weeks post-injury are shaded in orange and blue, respectively; orange indicates removal of CNO and suspension of treadmill training; blue indicates resumption of CNO administration (and continued absence of treadmill training).

### Immunohistochemistry

Animals were euthanized with overdoses of Fatal-Plus (Cat. No. V.P.L. 9373, Vortech, Dearborn, MI) and perfused intracardially with 4% paraformaldehyde. Using gross inspection, spinal cord from T8-T12 spinal segments (site of SCI), as well as DREADDs-injected DRG (L2-L5) and corresponding lumbar spinal cord, were dissected and post-fixed for 24–48 h (4°C). Tissue was then transferred to 30% sucrose in phosphate-buffered saline (PBS) for 3–5 days. All tissue was embedded in OCT cryostat sectioning medium, sectioned using a cryostat and immediately affixed to Colorfrost Plus microscope slides (Cat. No. 12-550-18, Fisher Scientific, Hampton, NH).

Lesion sizes (20 μm sections) were assessed with Hematoxylin and Eosin (H&E) staining. Prior to staining, slides were incubated in 4% paraformaldehyde in phosphate buffered solution (PBS) and PBS and then placed on a slide warmer to better adhere tissue sections to slides. Tissue was stained with H&E using methods from the manufacturer (Cat. No. 54348, ScyTek Inc., Logan, Utah). In brief, sections were washed with xylene and rehydrated with a series of decreasing ethanol concentrations ranging from 100 to 70%. Sections were rinsed with deionized water, stained with hematoxylin, washed with acid alcohol, and stained with eosin Y. A series of 95% and 100% ethanol concentrations was used to dehydrate the tissue, followed by xylene and Citrisolv washes. Slides were coverslipped with Cytoseal 60 (Cat. No. 8310-4, Thermo Fisher Scientific, Waltham, MA) and then left to dry under the fume hood.

DRG (10 μm sections) and corresponding lumbar spinal cord (20 μm sections) were thrice washed with phosphate-buffered saline tween (PBS-T). For amplification of mCherry signal, sections were incubated with dsRed primary antibody (rabbit, polyclonal; Cat. No. 632496, Takara Bio Inc., Mountain View, CA, RRID:AB_10013483) at 1:400 overnight at 4 deg C. The following day, sections were washed 5 times with PBS-T and incubated with Alexa Fluor 594 secondary antibody (donkey anti-rabbit; Cat. No. 111-585-144, Jackson Immunoresearch Laboratories Inc., West Grove, PA, RRID:AB_2307325) at 1:400 for 2 h at room temperature. Sections were again washed 5 times with PBS-T. Slides were then air-died and cover-slipped with Fluoromount-G (Cat. No. 0100-01, VWR International, Radnor, PA). With methods provided by the manufacturer, Fluorescent Nissl NeuroTrace, Cat. No. N21479, ThermoFisher Scientific, Waltham, MA) at 1:400 was used to visualize all neurons of the DRG.

Images of fluorescent tissue were acquired using a Zeiss microscope (Jena, Germany) at 10x magnitude. Sections with H&E stain were imaged using a Nikon Eclipse 80i microscope (Melville, NY) at 4x magnitude. Images of the same tissue were stitched together using Adobe Photoshop.

#### Quantification

Transduction efficiency of hM3Dq DREADDs within an injected DRG was quantified using adjacent DRG sections 10 μm apart stained with dsRed or Fluorescent Nissl. dsRed and Fluorescent Nissl positive cells were counted using Cell Counter on ImageJ. The fraction of dsRed (hM3Dq DREADDs) positive cells to total neurons (Fluorescent Nissl positive cells) was calculated for each injected DRG. Since transduction efficiency of each DRG is separate and is not influenced by other DRG that have been injected with DREADDs, we considered each DRG its own N in our statistical analyses. The sizes of transduced cells (dsRed+) were calculated by measuring the diameter of the cell bodies using ImageJ and are displayed as a histogram (size data are from an earlier cohort of rats using the same viral construct and surgical procedure). Healthy spared tissue was quantified using the NIS-Elements Basic Research program (Nikon, 2011). At the epicenter of the hemisection SCI of coronal sections, custom regions of interest (ROIs) were generated for individual animals. ROIs were converted from pixels^2^ to mm^2^ with scale bars that were annotated on images at the time of capture on the Nikon Eclipse 80i microscope.

Axonal projections of neurons transfected with AAV2-mCh (controls) or AAV2-hM3Dq DREADD-mCh are visible under fluorescent microscopy. Five lumbar spinal cord segments L2-L5 from each animal with detectable axonal fluorescence were analyzed for mCherry axonal density. Each section was at least 300 μm apart. Axon densities were quantified using the NIS-Elements Basic Research program. Two ROIs were defined—-the motor pools and Clarke's column, which were identified by comparison with a rat spinal cord atlas (Tang et al., 2007; Watson et al., 2009; Kelamangalath et al., 2015). Clarke's column was identified by location and density of innervation. We used an atlas to define the area and verified by vGLUT1 staining (data not shown). Similarly, the shape of the ventral horn was encircled using the atlas in laminas 8 and 9. For each ROI, a threshold was set to eliminate background autofluorescence. mCherry fluorescence was determined as a fraction of the total area of the ROI.

### Statistical analysis

Outcome measures from several histological calculations are proportional data, e.g., resultant values lie between 0 and 100 percent. Proportional data may skew the variance and risk invalidating the evaluation of statistical significance (Sokal and Rohlf, 1995). As such, we removed the variance from the means of these data by applying an arcsine transformation.

We used linear mixed effects models [with the *nlme* package in *R*; (R Core Team, 2021)] to analyze our aggregated kinematic data, because these models are well-suited to handling hierarchical, repeated measures data, and data sets that may contain missing or unequal cases, which are typically encountered in spinal cord injury studies.

The kinematic parameters in **Figures 3A–I**, **4B–F**, **5C** were analyzed as follows. The final dependent variables input into each model were computed from the maximum, minimum, range, mean (**Figures 3A–E,G–I**, **4B**, **5C**) or standard deviation (**Figures 3F**, **4C–F**) of each parameter across time points within each stride, followed by the mean of these by stride values for each unique grouping of rat, time point, and speed. This resulted in one data point per rat in each unique combination of rat, time point, and speed. Individual rats were assigned to either control or experimental treatments. These data were examined for normality within each group with the Shapiro-Wilk test, and were not found to be significantly different from normal. To be conservative, however, non-parametric Wilcoxon Rank Sum tests were still used for *post-hoc* analyses within time points.

The linear mixed effects model fit to these data included fixed effect terms for (1) treatment (having two levels: excitatory DREADDs activated by CNO, hereafter called the “DREADDs” group, and the control group, consisting of administration of CNO to animals with sham-mCherry construct injections, hereafter called “CNO”) and (2) time point (having seven levels from pre-injury to 8 weeks post injury). It further included a constant random effect term for rat. To test for the necessity of random effects, models with and without additional levels of nested random effects were fit and compared with the Akaike information criterion; random effects were added until this value was no longer decreased by a value of 2 or more. In this manner we found that nested random effects for time point or speed group did not improve the model, and were omitted. The resulting model structure captures the fixed effects whilst accounting for the hierarchical repeated measures taken at multiple time points within each rat. The significance of main effects and interactions in each model were evaluated using an analysis of variance (ANOVA; *anova.lme* function). Where models had a significant main effect for treatment or a significant interaction between treatment and time point (*p* < 0.05), *post hoc* comparisons between data at each time point were made with the Wilcoxon Rank Sum test (also deemed significant at *p* < 0.05; *wilcox_test* function). Since the effect of time point is not our focus but rather the treatment (Except in **Figure 5C**), we do not report its significance for the parameters displayed in the Figure panels listed above. Typically, it was significant due to large changes with the injury and on recovery for these data.

The time series data in **Figure 4A** were computed as follows. Ankle joint angle data were first averaged across strides at each percent stance bin within each unique grouping of rat, time point, and speed, resulting in one average time series for each rat in each condition (time point and speed). The mean and standard error of the mean across rats was then computed separately for rats within each treatment category (DREADDs or control), as a function of percent stride bin, resulting in the plot in **Figure 4A**. A Wilcoxon Rank Sum test was carried out at each percent stance between DREADDs and controls groups, and the significant (*p* < 0.05) time points are noted by the black bar at the bottom of the figure.

The buckling data in **Figure 5C** with dependent variable *Range ASIS Height* were analyzed with a linear mixed effects model having fixed effects for time point and speed group, and a constant random effect by time point nested within rat. Significance of fixed effects were evaluated as above using the *anova.lme* function, and *post-hoc* tests between time points were then computed using estimated marginal means [*R emmeans* function; (Searle et al., 1980)].

## Results

**Figure 1:**
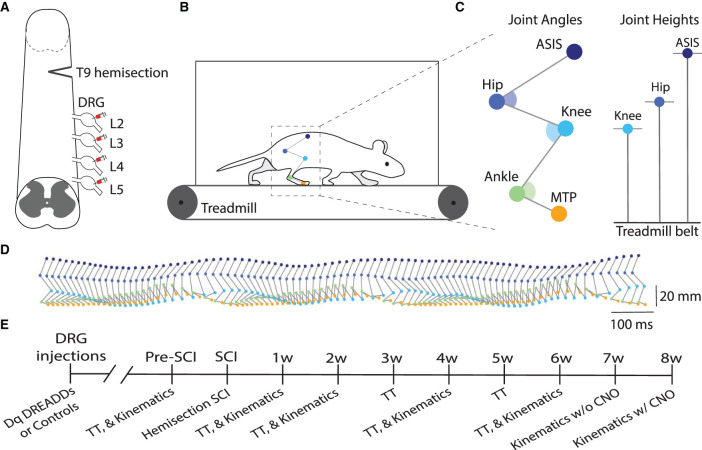
DRG injections and hindlimb kinematics. **(A)** Illustrative schematic of hM3Dq (excitatory) DREADDs injection into the lumbar (L2-L5) dorsal root ganglia (DRG) and right-side hemisection injury at T9. **(B)** Five hindlimb joints were labeled and filmed at 250 FPS during treadmill locomotion at 5 speeds (16, 20, 24, 28, and 32 cm/s). Pose estimation of joint trajectories were extracted with DeepLabCut and then reconstructed in 3D with a custom program. **(C)** The following five joint locations were tracked: ASIS (anterior superior iliac spine), hip, knee, ankle, and MTP (metatarsophalangeal) joints. Example kinematic variables include joint angles and joint heights. **(D)** Side view of hindlimb kinematics during treadmill locomotion. **(E)** Experimental timeline of study (SCI, spinal cord injury; TT, treadmill training).

Animals were injected with hM3Dq DREADDs or control virus in lumbar (L2-L5) DRG and received a right-side T9 hemisection SCI (Figure 1A). Figures 1B–D present illustrative schematics of hindlimb kinematics acquisition and analysis. Five hindlimb joints/points were labeled: ASIS (anterior superior iliac spine), hip, knee, ankle, and MTP (metatarsophalangeal). Recordings were obtained at 250 FPS during treadmill locomotion at 5 speeds (16, 20, 24, 28, and 32 cm/s). Estimation of joint positions were extracted with DeepLabCut (Mathis et al., 2018; Nath et al., 2019) and then reconstructed in 3D (Maghsoudi et al., 2019). An experimental timeline is presented in Figure 1E: kinematics were recorded pre-injury, and 1, 2, 4, and 6 weeks post-injury; and treadmill training was conducted thrice weekly.

### mCherry positive axonal densities in the lumbar spinal cord

**Figure 2:**
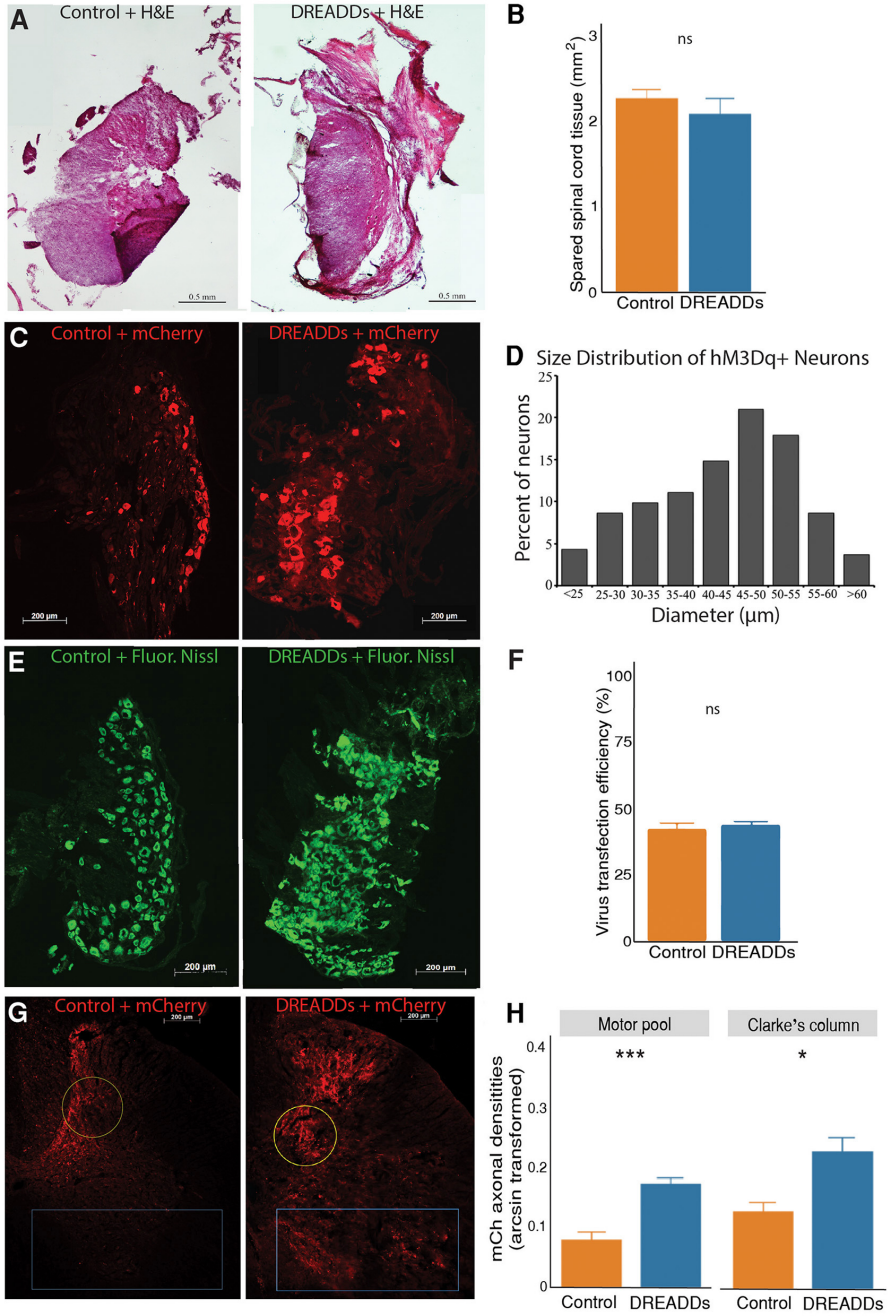
Activation of targeted afferents by hM3Dq DREADDs increases afferent plasticity in the lumbar spinal cord. **(A)** Extent of hemisection injury was measured at the epicenter of the injury with H&E staining. **(B)** To examine lesion size between the control and DREADDs groups, we compared spared tissue at the epicenter of the hemisection. We did not observe a statistically significant difference between healthy spared tissue between groups (controls = 2.26 ± 0.11 mm^2^; *n* = 6; DREADDs animals = 2.07 ± 0.18 mm^2^; *n* = 8; mean ± SEM; *p* = 0.39; *t*-test). **(C)** DRG injected with control AAV virus and virus with hM3Dq DREADDs. **(D)** Histogram of diameter sizes of transduced DRG cells. **(E)** Fluorescent Nissl stain DRG sections, 10 μm apart from sections in. **(C)** To measure transduction of efficiencies of viral vectors, the fraction of transduced cells (dsRed+) to total DRG cells (Fluorescent Nissl+) was calculated. **(F)** Transduction efficiencies of viral vectors (AAV with and without DREADDs) were not significantly different between groups (controls = 42% ± 2.5%; *n* = 12; DREADDs animals = 44% ± 1.4%; *n* = 10; mean ± SEM; *p* = 0.64; *t*-test). **(G)** Lumbar spinal cord from injected DRGs of control and DREADDs animals. **(H)** We observed a significantly higher density of mCherry axons in DREADDs animals within the motor pools (blue rectangle; controls = 0.08 ± 0.01; *n* = 4; DREADDs animals = 0.17 ± 0.01; *n* = 6; mean ± SEM of arcsine transformed data; *p* = 0.00053; *t*-test) and within Clarke's column (yellow circle; controls = 0.13 ± 0.02; *n* = 4; DREADDs animals = 0.23 ± 0.02; *n* = 6; mean ± SEM of arcsine transformed data; *p* = 0.021; *t*-test). *, *p* < 0.05; **, *p* < 0.01; ***, *p* < 0.001.

Spared tissue was considered healthy if it was intact and had uniform H&E-stained color (Figure 2A). The right hemicord was removed, including the dorsal and ventral horns. Spared tissue consisted of the entirety of the left hemicord, including the dorsal and ventral horns, and indicates healthy tissue that is undamaged by the injury. We did not observe significant differences in healthy spared tissue between groups (Figure 2B; controls = 2.26 ± 0.11 mm^2^; *n* = 6; DREADDs animals = 2.07 ± 0.18 mm^2^; *n* = 8; mean ± SEM; *p* = 0.39; *t*-test).

In this study, we injected right lumbar DRG L2-L5 with either excitatory (hM3Dq) DREADDs or control virus (Figure 1A). Neurons expressing mCherry were observed in DRG of controls and DREADDs animals (Figure 2C). Diameter sizes of transduced afferents are presented in a histogram in Figure 2D, with preferential targeting occurring in cells that are medium and large in size (Li and Zhao, 1998; Liu et al., 2002). To calculate transduction efficiencies of viral vectors (fraction of dsRed+ cells to total cells in adjacent sections), Fluorescent Nissl was used to visualize total cells in the DRG (Figure 2E). Transduction efficiencies were not significantly different between groups (Figure 2F; controls = 42% ± 2.5%; *n* = 12; DREADDs animals = 44% ± 1.4%; *n* = 10; mean ± SEM; *p* = 0.64; *t*-test). Axons of transduced neurons were observed in the lumbar spinal cord of controls and DREADDs animals (Figure 2G). Sections show that mCherry fluorescent axons are present in multiple laminae, including the lamina of the ventral horn (including the motor pools) and lamina VII (including Clarke's column). Density of mCherry axons in the motor pools and Clarke's column were compared between groups (Figure 2H). We report a significantly higher density of mCherry axons in the motor pools of DREADDs animals (controls = 0.08 ± 0.01; *n* = 4; DREADDs animals = 0.17 ± 0.01; *n* = 6; mean ± SEM of arcsine transformed data; *p* = 0.00053; *t*-test). Additionally, DREADDs animals show significantly higher density of mCherry axons in the Clarke's column (controls = 0.13 ± 0.02; *n* = 4; DREADDs animals = 0.23 ± 0.02; *n* = 6; mean ± SEM of arcsine transformed data; *p* = 0.021; *t*-test).

### Behavioral and kinematics differences

**Figure 3:**
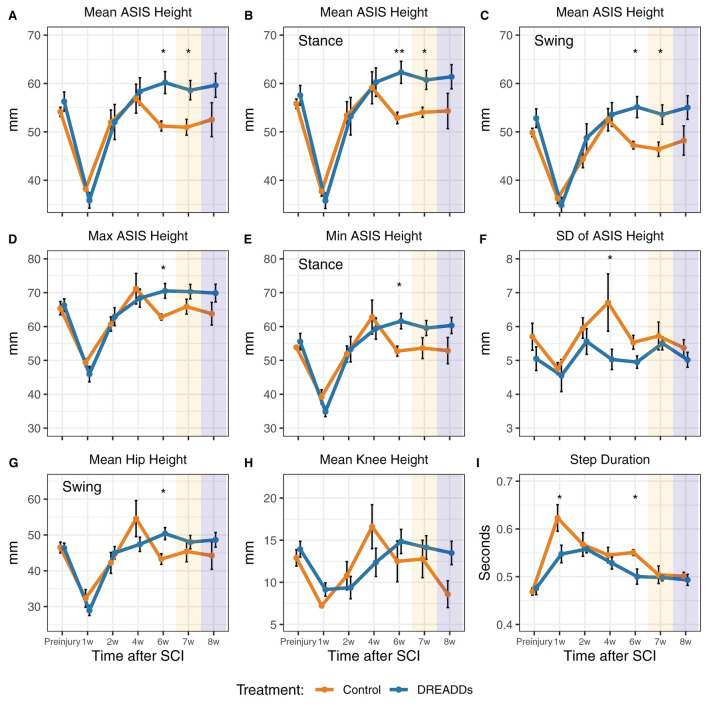
Afferent excitation by DREADDs leads to higher height and more stability of the hindquarters. Parameters of hindlimb landmarks were extrapolated using DeepLabCut, a custom program for 3D reconstruction, and subsequent analyses. Landmarks include the anterior superior iliac spine (ASIS), hip joint, knee joint, ankle joint, and metatarsophalangeal (MTP) joint. **(A)** In the overall stride, we observed significantly higher ASIS heights in the DREADDs group [16 cm/s: treatment x time interaction significant *F*_(6, 64)_ = 3.44; *p* = 0.005; ANOVA on linear mixed effects model (LME), and hereafter unless noted; *post-hoc* week 6: controls = 51.2 ± 1.0 mm; *n* = 6; DREADDs animals = 60.2 ± 2.3 mm; *n* = 8; mean ± SEM; *p* = 0.011; Wilcoxon Rank sum test, and hereafter for *post-hoc* tests unless noted; furthermore, means, SEM, and sample sizes for control and DREADDs animals are the same hereafter unless noted; *post-hoc* week 7: controls = 50.9 ± 1.7 mm; DREADDs animals = 58.6 ± 2.0 mm; *p* = 0.020]. **(B)** In the stance phase, we observed higher ASIS joints of DREADDs animals [16 cm/s, treatment x time significant *F*_(6, 65)_ = 2.53; *p* = 0.029; *post-hoc* week 6: controls = 52.9 ± 1.2 mm; DREADDs animals = 62.3 ± 2.3 mm; *p* = 0.006; *post-hoc* week 7: controls = 54.1 ± 1.0 mm; DREADDs animals = 60.7 ± 2.0 mm; *p* = 0.016]. **(C)** In the swing phase, DREADDDs exhibited significantly higher ASIS joint heights [16 cm/s: treatment x time interaction significant *F*_(6, 66)_ = 3.19; *p* = 0.008; *post-hoc* week 6: controls = 47.2 ± 0.8 mm; DREADDs animals = 55.1 ± 2.2 mm; *p* = 0.011; *post-hoc* week 7: controls = 46.4 ± 1.5 mm; DREADDs animals = 53.6 ± 2.0 mm; *p* = 0.011]. **(D,E)** The DREADDs group displayed a significantly higher extremes for ASIS height [**D**; max; 16 cm/s: treatment x time interaction significant *F*_(6, 65)_ = 2.52; *p* = 0.03; *post-hoc* week 6: controls = 62.8 ± 0.9 mm; DREADDs animals = 70.5 ± 2.2 mm; *p* = 0.011; **E**; min; 16 cm/s: treatment x time interaction significant *F*_(6, 68)_ = 2.60; *p* = 0.025; *post-hoc* week 6: controls = 53.6 ± 3.1 mm; DREADDs animals = 61.6 ± 2.3 mm; *p* = 0.019]. **(F)** We observed a smaller standard deviation of ASIS heights in the DREADDs animals [16 cm/s: main effect of treatment significant *F*_(1, 12)_ = 8.93; *p* = 0.011; *post-hoc* week 4: controls = 6.71 ± 0.85 mm; DREADDs animals = 5.03 ± 0.3 mm; *p* = 0.045]. **(G)** We observed significantly higher hip heights in the swing phase in DREADDs animals [16 cm/s: treatment x time interaction significant *F*_(6, 68)_ = 2.86; *p* = 0.015; *post-hoc* week 6: controls = 43.3 ± 1.5 mm; DREADDs animals = 50.4 ± 1.7 mm; *p* = 0.01]. **(H)** While we observed a significant interaction effect between treatment and time point in mean knee height, we did not find a significant difference in the Wilcoxon Rank Sum *post-hoc* tests [16 cm/s: treatment x time interaction significant *F*_(6, 68)_ = 2.81; *p* = 0.017]. **(I)** Duration of step cycles was observed to be significantly shorter in the DREADDs group [24 cm/s; main effect of treatment significant *F*_(1, 12)_ = 5.25; *p* = 0.041; *post-hoc* week 1: controls = 0.623 ± 0.028 s; DREADDs animals = 0.548 ± 0.018 s; *p* = 0.035; *post-hoc* week 6: controls = 0.551 ± 0.006 s; DREADDs animals = 0.500 ± 0.016 s; *p* = 0.019]. Treadmill training was terminated after 6 weeks post-SCI. Locomotion was then recorded in the absence of CNO (7w; orange shaded region) and then again with CNO to control for history effects (8w; blue shaded region). *, *p* < 0.05; **, *p* < 0.01.

**Figure 4:**
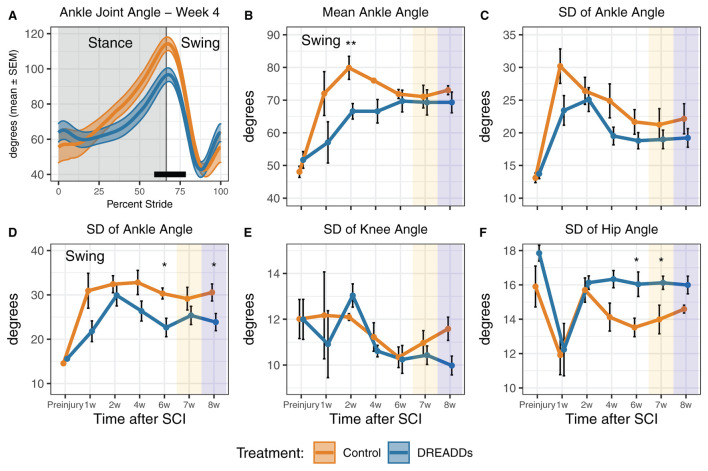
DREADDs activation promote joint angles and joint angle variability that are closer to the preinjury condition. **(A)** Significant differences in percent stride between groups were observed, particularly in the phase transition from stance to swing (24 cm/s; week 4: 59–78.5% stride significant; *t*-test and Wilcoxon Rank Sum *post-hoc* test). **(B)** The DREADDs group displayed significantly less flexed ankle angles in the swing phase [24 cm/s: treatment x time interaction significant *F*_(6, 64)_ = 2.42; *p* = 0.036; ANOVA on linear mixed effects model (LME), and hereafter unless noted; *post-hoc* week 2: controls = 79.9 ± 3.54 deg; DREADDs animals = 66.6 ± 2.38 deg; *p* = 0.005; Wilcoxon Rank sum test, and hereafter for *post-hoc* tests unless noted; furthermore, means, SEM, and sample sizes for control and DREADDs animals are the same hereafter unless noted]. **(C)** The standard deviation of each joint angle was compared between groups to investigate differences in the range of motion of each joint as it moved through a stride and during swing/stance phases. We did not observe significant difference in standard deviation in ankle joint angle between groups. **(D)** We did, however, observe that the DREADDs group had significantly less standard deviation in ankle joint angle in the swing phase [16 cm/s: main effect of treatment significant *F*_(1, 12)_ = 7.85; *p* = 0.016; *post-hoc* week 6: controls = 30.3 ± 1.25 deg; DREADDs animals = 22.6 ± 2.07 deg; *p* = 0.019; *post-hoc* week 8: controls = 30.5 ± 1.92 deg; DREADDs animals = 23.9 ± 1.94 deg; *p* = 0.043]. **(E)** We did not observe significant difference in standard deviation in knee joint angle between groups. **(F)** The DREADDs group exhibited significantly larger standard deviation in hip joint angle [**F**; 24 cm/s: main effect of treatment significant *F*_(1, 12)_ = 4.87; *p* = 0.048; *post-hoc* week 4: controls = 14.1 ± 0.8 deg; DREADDs animals = 16.3 ± 0.5 deg; *p* = 0.059; *post-hoc* week 6: controls = 13.5 ± 0.5 deg; DREADDs animals = 16.0 ± 0.7 deg; *p* = 0.02; *post-hoc* week 8: controls = 14.6 ± 0.2 deg; DREADDs animals = 16.0 ± 0.5 deg; *p* = 0.02]. Treadmill training concluded after 6 weeks post-SCI. Locomotion was then recorded in the absence of DREADDs activator CNO (7w; orange shaded region) and then again with CNO to control for history effects (8w; blue shaded region). *, *p* < 0.05; **, *p* < 0.01.

**Figure 5:**
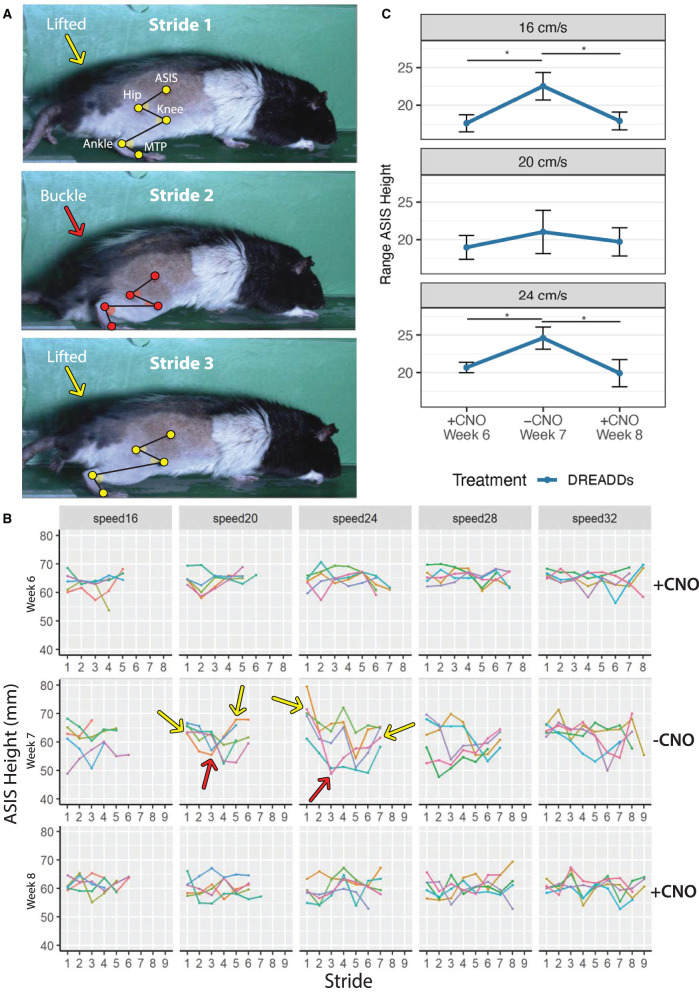
A buckling phenomenon occurs in the absence of DREADDs activation. Animals were subjected to run in the absence of CNO during Week 7 (−CNO). Results were compared to running bouts with CNO administration (Weeks 6 and 8; +CNO). Triweekly treadmill training was discontinued between week 6 through week 8 to prevent progression of recovery due to exercise training. Only results for DREADDs animals are shown as control animals did not exhibit the buckling event in the absence of CNO. **(A)** Grossly observed buckling phenomenon (occurred in Stride 2). DREADDs animals would take one to several normal strides; suddenly drop their hindquarters onto the treadmill belt and remain seated on the belt moving their hindlimb as if they were mid-stride, but unable to stand (buckling event); posturally correct their error by lifting their hindquarters off the belt; and begin locomoting normally again. Hindlimb landmarks (ASIS joint, hip joint, knee joint, ankle joint, and MTP) exhibit a change in hindquarter height between strides. **(B)** ASIS joint heights for one rat. Heights were stable with +CNO and instable with −CNO. Dips in ASIS joint height with −CNO correspond to the buckling events when the hindquarters dropped onto the belt. Arrows indicate sample buckling events with lifted hindquarters in yellow and buckling events in red. **(C)** DREADDs animals exhibited a significantly larger ASIS height range across speeds [linear mixed effects (LME) model with fixed effects for time point and speed; random effect for rat and time point nested within rat; main effect of timepoint significant *F*_(2, 13)_ = 6.12; *p* = 0.013; ANOVA on LME]. We observed a significantly larger ASIS height range at 16 cm/s (top; +CNO, week 6 = 17.6 ± 1.1 mm; −CNO, week 7 = 22.5 ± 1.8 mm; +CNO, week 8 = 17.9 ± 1.2 mm; *n* = 8; mean ± SEM; *post-hoc* +CNO, week 6 to −CNO, week 7: *p* = 0.007; Wilcoxon Rank sum test, and hereafter for *post-hoc* tests unless noted; furthermore, means, SEM, and sample sizes for control and DREADDs animals are the same hereafter unless noted; *post-hoc* −CNO, week 7 to +CNO, week 8: *p* = 0.007). We noted a trend toward larger ranges at 20 cm/s (middle; +CNO, week 6 = 19.0 ± 1.6 mm; −CNO, week 7 = 21.0 ± 1.9 mm; +CNO, week 8 = 19.7 ± 1.9 mm; *post-hoc* +CNO, week 6 to −CNO, week 7: *p* = 0.145; *post-hoc* −CNO, week 7 to +CNO, week 8: *p* = 0.214). Finally, we observed a significantly larger range at 24 cm/s (bottom; +CNO, week 6 = 20.7 ± 0.7 mm; −CNO, week 7 = 24.6 ± 1.5 mm; +CNO, week 8 = 19.9 ± 1.8 mm; *post-hoc* +CNO, week 6 to −CNO, week 7: *p* = 0.015; *post-hoc* −CNO, week 7 to +CNO, week 8: *p* = 0.011). *, *p* < 0.05.

In Figures 3–5, we present kinematics data exhibited by animals with excitatory DREADDs and controls. All animals were exercised trained for 6 weeks post-SCI. To determine if CNO is required to see locomotor changes observed with DREADDs activation, we recorded treadmill locomotion in the absence of CNO (denoted as “7w” or week 7 in Figures 3, 4 and “−CNO, Week 7” in Figure 5). In the following week, we recorded treadmill locomotion with CNO administration to control for history effects (denoted as “8w” or week 8 in Figures 3, 4 and “+CNO, Week 8” in Figure 5). In Figures 3, 4, kinematics graphs are shaded with orange and blue over 7 and 8 weeks post-injury, respectively.

In accordance with Tukey's rule (Tukey, 1977; Hoaglin, 2003), for all kinematics data, values outside 1.5 times the interquartile range of the respective feature, week, and speed were omitted from the following analyses. Strides were examined as a whole as well as broken into swing and stance phases. Low speeds include 16 and 20 cm/s, the mid-range speed was 24 cm/s, and high speeds include 28 and 32 cm/s. Five joints in the right hindlimb were examined for their joint/limb kinematics: anterior superior iliac spine (ASIS), hip, knee, ankle, and metatarsophalangeal (MTP) joints. Table 1 presents statistical values for the plots in Figures 3, 4 and summarizes the following results. In the later stages of recovery, we observed significantly higher ASIS heights in the DREADDs group in the overall stride (Figure 3A). In the stance phase, we observed higher ASIS joints of DREADDs animals (Figure 3B). Further, in the swing phase, DREADDDs also displayed significantly higher ASIS joint heights (Figure 3C). Additionally, the DREADDs group displayed a significantly higher max (Figure 3D) and min ASIS height (Figure 3E). We also observed a smaller standard deviation of ASIS heights in the DREADDs animals (Figure 3F). In DREADDs animals, we observed significantly higher hip heights in the swing phase (Figure 3G). While we observed a significant interaction effect between treatment and time point in mean knee height, we did not find a significant difference in the Wilcoxon Rank Sum *post-hoc* tests (Figure 3H). Lastly, DREADDs animals displayed significantly shorter step durations (Figure 3I).

**Table 1:** Summary of statistical analyses of effect of afferent activation on kinematics in SCI recovery.

			**Main effects**			***Post-hoc*** **tests**							
							**Control**			**DREADDs**			
**Variable**	**Phase**	**Speed (cm/s)**	**Significant term**	***F*-statistic**	***P***	**Week**	**Mean**	**SD**	***N***	**Mean**	**SD**	***N***	***P***
ASIS height	Whole	16	Treatment x time	*F*_(6, 64)_ = 3.44	0.005	6	51.2 mm	1.0 mm	6	60.2 mm	2.3 mm	8	0.011
						7	50.9 mm	1.7 mm	6	58.6 mm	2.0 mm	8	0.02
ASIS height	Stance	16	Treatment x time	*F*_(6, 65)_ = 2.53	0.029	6	52.9 mm	1.2 mm	6	62.3 mm	2.3 mm	8	0.006
						7	54.1 mm	1.0 mm	6	60.7 mm	2.0 mm	8	0.016
ASIS height	Swing	16	Treatment x time	*F*_(6, 66)_ = 3.19	0.008	6	47.2 mm	0.8 mm	6	55.1 mm	2.2 mm	8	0.011
						7	46.4 mm	1.5 mm	6	53.6 mm	2.0 mm	8	0.011
ASIS height max	Whole	16	Treatment x time	*F*_(6, 65)_ = 2.52	0.03	6	62.8 mm	0.9 mm	6	70.5 mm	2.2 mm	8	0.011
ASIS height min	Whole	16	Treatment x time	*F*_(6, 68)_ = 2.60	0.025	6	53.5 mm	3.1 mm	6	61.6 mm	2.3 mm	8	0.019
SD of ASIS height	Whole	16	Treatment	*F*_(1, 12)_ = 8.93	0.011	4	6.71 mm	0.85 mm	6	5.03 mm	0.3 mm	8	0.045
Hip height	Swing	16	Treatment x time	*F*_(6, 68)_ = 2.86	0.015	6	43.3 mm	1.5 mm	6	50.4 mm	1.7 mm	8	0.01
Knee height	Whole	16	Treatment x time	*F*_(6, 68)_ = 2.81	0.017				6			8	
Step duration	Whole	16	Treatment	*F*_(1, 12)_ = 5.25	0.041	1	0.623 s	0.028 s	6	0.548 s	0.018 s	8	0.035
						6	0.551 s	0.006 s	6	0.5 s	0.016 s	8	0.019
Ankle joint angle	Swing	24	Treatment x time	*F*_(6, 64)_ = 2.42	0.036	2	79.9 deg	3.54 deg	6	66.6 deg	2.38 deg	8	0.005
SD of ankle joint angle	Swing	16	Treatment	*F*_(1, 12)_ = 7.85	0.016	6	30.3 deg	1.92 deg	6	22.6 deg	2.07 deg	8	0.019
						8	30.5 deg	1.25 deg	6	23.9 deg	1.94 deg	8	0.043
SD of hip joint angle	Whole	24	Treatment	*F*_(1, 12)_ = 4.87	0.048	4	14.1 deg	0.8 deg	6	16 deg	0.5 deg	8	0.059
						6	13.5 deg	0.5 deg	6	16.3 deg	0.7 deg	8	0.02
						8	14.6 deg	0.2 deg	6	16 deg	0.5 deg	8	0.02

Presented are the variable modeled, which phase(s) of the stride were analyzed (stance, swing, or whole stride), followed by the main effects, including which term of the linear mixed effects model was significant (treatment, or the interaction treatment x time; although the time term was typically significant, we do not report it here as it is expected to vary with injury) and associated F-statistic and P-value; finally, the results of *post-hoc* testing are shown, giving the week post-injury, and the mean, SD, N, and P-values for each comparison. Linear mixed effects models implemented with the R nlme package and Wilcoxon Rank sum tests; see Methods for full details of the statistical modeling and Figures 3, 4 and the Results and Discussion sections for interpretation.

Table 1 further presents statistical values for the plots seen in Figure 4. Figure 4A shows significant differences in ankle joint angle vs. percent stride between treatment groups. We observed a significantly more flexed ankle angle in the swing phase in the DREADDs group (Figure 4B). We then used standard deviation as a measure of the range of motion that each joint moves through during a stride or during swing/stance phases. We compared the standard deviation of each joint angle to investigate differences in range of motion of joints between groups. We did not observe significant differences in standard deviation in ankle joint angle between groups (Figure 4C). However, we did observe that the DREADDs animals had significantly lower standard deviation in ankle joint angle in the swing phase (Figure 4D). We did not observe a significant difference in standard deviation in knee joint angle between groups (Figure 4E). Finally, DREADDs animals exhibited significantly larger standard deviation of hip joint angle (Figure 4F).

### Withdrawal of CNO in animals with excitatory DREADDs

We observed a buckling phenomenon, or collapse of the hindquarters, in DREADDs animals in the absence of CNO (−CNO). During the event, animals would locomote normally, suddenly drop their hindquarters on the treadmill belt, and within an additional one to two strides, they would posturally correct their error and lift their hindquarters to locomote normally again (Figure 5A; buckling event happened in Stride 2 indicated by orange arrow). Sometimes this would be followed by a second buckling event within the same trial. Buckling was not observed in any control animals. Figure 5B presents the height of the ASIS for one animal with +CNO (top, week 6), −CNO (middle, week 7, CNO withdrawal), and +CNO again (bottom, week 8). Line plots represent trials (e.g., 5 trials per speed) and points represent strides. Sample buckling events are shown with arrows: lifted hindquarters in yellow and buckling events in red. In comparison to +CNO (week 6), the ASIS joint height with −CNO (week 7) appears more unstable, with dips in ASIS height corresponding to the buckling events where the hindquarters dropped onto the belt. The ASIS joint returned to a more stable height in the following week with +CNO (week 8), albeit slightly less stable than in week 6.

Quantifying this phenomenon was difficult as the event can be masked by normal strides that flank the buckling event. To uncover it, we looked at ASIS height range (maximum minus minimum ASIS height) within trials and then averaged them across speeds to avoid pseudoreplication. The buckling event would putatively present itself as a larger range in ASIS heights (Table 2). We observed a significantly larger ASIS height range for DREADDs animals across speeds (Figure 5C). Within speeds, we observed a significantly larger ASIS height range at 16 cm/s, a trend toward larger ranges at 20 cm/s, and a significantly larger range at 24 cm/s.

**Table 2:** Summary of statistical analyses of the buckling effect.

**Variable**	**Speed (cm/s)**	**Condition**	**Mean**	**Std. error**	***N***	**Interaction**	***P*-value**
		+CNO, week 6	17.6 mm	1.1 mm		+CNO, week 6 to −CNO, week 7 −CNO, week 7 to +CNO, week 8	0.007 0.007
ASIS height range	16	−CNO, week 7	22.5 mm	1.8 mm	8		
		+CNO, week 8	17.9 mm	1.2 mm			
		+CNO, week 6	19.0 mm	1.6 mm		+CNO, week 6 to −CNO, week 7 −CNO, week 7 to +CNO, week 8	0.145 0.214
ASIS height range	20	−CNO, week 7	21.0 mm	1.9 mm	8		
		+CNO, week 8	19.7 mm	1.9 mm			
		+CNO, week 6	20.7 mm	0.7 mm		+CNO, week 6 to −CNO, week 7 −CNO, week 7 to +CNO, week 8	0.015 0.011
ASIS Height Range	24	−CNO, week 7	24.6 mm	1.5 mm	8		
		+CNO, week 8	19.9 mm	1.8 mm			

Presented are the variable modeled, the speed of treadmill locomotion, the condition (time point post injury and CNO presence/absence during that time point), the mean and SD of Range ASIS height range in mm, the N value, and the P-value of the listed interactions between conditions (time point and CNO presence/absence). CNO was administered at week 6, withdrawn in week 7, and re-administered in week 8. DREADDs animals exhibited a significantly larger ASIS height range in week 7 across speeds. Linear mixed effects (LME) models with fixed effects for time point/CNO and speed, and a random effect for rat and time point/CNO nested within rat. The main effect of timepoint/CNO was significant *F*_(2, 13)_ = 6.12; *p* = 0.013; ANOVA on LME, with results for the specific *post-hoc* interactions (Wilcoxon Rank Sum tests) are shown in the last column. See Figure 5 and Results and Discussion for interpretation.

### Dependence of kinematic variables on lesion spared tissue area and DRG transduction efficiency

Mean values of kinematic variables (one value per animal) at 6 weeks post injury and speed 16 cm/s were fit against lesion spared tissue remaining (mm^2^) and DRG transduction efficiency (percent of neurons) using the R *lm* function (Supplementary Table S1; Supplementary Figures S3, S4). The estimate of the slope (“Estimate”) term of the model for each variable is displayed, with associated metrics of the model fit and significance. None of the models against spared tissue approach significance, suggesting the lesion size was not causing significant variation in these kinematic variables.

For DRG transduction efficiency, only standard deviation of ankle angle during swing was statistically significant, while standard deviation of ASIS height was close to significant. Note that these results (for DRG transduction efficiency) are within excitatory DREADs animals only, and support the conclusion that the DREADDs excitation is causing the observed changes in kinematics seen in Figures 3–5. This variable (*SD Ankle Angle Swing*) was not significant in the control group, as expected as these animals did not have excitatory DREADDs.

### Summary of results

To return to the questions that motivated our study, the differences in kinematics between control and DREADDs excited animals and the buckling behavior we observe support a positive response to our first question: activation of large diameter peripheral afferents with hM3Dq DREADDs does influence recovery from a hemisection spinal cord injury in the rat model. For the latter aim, to begin to uncover underlying mechanisms of plasticity, we examined neuroanatomical changes in the afferents we have modulated in the Clarke's column and motor pools. While we note that observed morphological changes do not allow us to definitively conclude that functional plasticity occurring within these circuits, results of cFOS expression support that DREADDs are indeed promoting activation of modulated pathways.

## Discussion

Advances in therapeutic interventions for individuals with SCI, such as epidural electrical stimulation (EES), have demonstrated the ability to mediate functional improvements putatively *via* the rewiring damaged and spared circuitry. Uncovering the neural circuit changes involved in promoting functional recovery is a critical challenge for the field, and while electrical spinal cord stimulation modalities are robust, modern genetic tools offer advantages in deterministically tracing affected circuits and quantifying plasticity. In this study, we sought to uncover some of the mechanisms that may underlie rehabilitation with excitation of large diameter afferents, and describe concomitant changes in locomotion with detailed kinematics. With our transduction viral approach, we coupled hM3Dq (excitatory) DREADDs expression with a fluorescent protein for immunohistological characterization of neural circuit changes involved with activation of targeted lumbar afferents.

It is well-accepted that synaptic plasticity plays an important role in behavioral improvements after SCI (Waters et al., 1996; Burns et al., 1997). In this study, we report larger densities of mCherry fluorescence in the DREADDs group in both the motor pools and Clarke's column (Figure 2). Given that we observe similar spared tissue at the epicenter of the hemisection (Kloos et al., 2005) and AAV2 transduction rates in the DRG of both groups, our results suggest that DREADDs activation may have induced plasticity of targeted afferents onto interneurons and motorneurons.

Axonal sprouting and synaptogenesis within motor pools can directly and indirectly drive muscle activity by supplementing motorneuron activation after SCI. Directly, plasticity within monosynaptic connections of group Ia afferents onto motorneurons that occur within the same muscle target could be influential in regaining of volitional control of affected muscles (Eccles et al., 1957; Takeoka et al., 2014; Moraud et al., 2016; Takeoka, 2019; Takeoka and Arber, 2019; Eisdorfer et al., 2020). Plasticity in the lumbar spinal cord promoted by hM3Dq DREADDs in multiple muscles within the same extensor or flexor group may support an increase in overall muscle force generated as indicated by the lifted and potentially more stable hindquarters seen in Figure 3. The evidence suggests that chronic DREADDs activation of afferents is inducing behavioral differences, potentially mediated by the plasticity observed in histology. DREADDs activation may cause stiffer hindlimb musculature by co-activating extensors and flexors. It may also extend the limb by activating more extensors than flexors, or simply because extensor musculature and/or moment arms are larger (Latash, 2018). With stiffer muscles and a potential bias toward extension, DREADDs animals may be capable of lifting their hindquarters higher than their control counterparts. Neuromechanical models of afferent excitation in moving rodents have suggested that increasing afferent excitation results in a more extended limb (S. Danner, *personal communication*; Danner et al., 2017). Interestingly, DREADDs animals exhibit a more flexed ankle joint angle during the transition between stance to swing (Figure 4A) and during the swing phase (Figure 4B) at intermediate time points. Afferent activation by DREADDs may promote more appropriate ankle joint movements during the end of the stance phase, aiding in push-off and reducing toe-drags upon liftoff.

Indirectly, plasticity may also occur where modulated afferents synapse onto interneuronal circuitry that conveys information to the motor pools, such as interneurons involved in lumbar central pattern generators (CPGs). Plasticity onto CPG circuitry may aid in adapting to perturbations and transmission of rhythmic activity for hindlimb coordination after injury (Cowley et al., 2008; Bui et al., 2013, 2016; Young, 2015; Danner et al., 2017; Laliberte et al., 2019; Shepard et al., 2021; Zholudeva et al., 2021), which may underlie observed step durations that are closer to the pre-injury condition in DREADDs animals as compared to their control counterparts at intermediate times (Figure 3I). In theory, activation by hM3Dq DREADDs in muscles about the hip could serve to promote hindlimb coordination by helping to control phase transitions and entrain rhythmic flexor and extensor activation (Andersson and Grillner, 1983; Kriellaars et al., 1994; Hiebert et al., 1996; Kiehn, 2006; Onushko, 2009). This may seem counterintuitive given the tonic nature of DREADDs activation, but tonic EES is thought to promote phase-appropriate changes in muscle activity, for appropriate levels of stimulation, due in part to the filtering properties of the CPG circuitry (Moraud et al., 2016). If true, afferents expressing hM3Dq DREADDs may be influential in the oscillating activation of neurons located within lumbar CPGs. Further studies could more directly tie this observation by gathering information from muscle recordings during locomotion bouts.

The aforementioned kinematic variables that were significantly different in DREADDs activated animals at intermediate time points, and then regressed to match the controls, could reflect plasticity and motor learning at these time points to handle the nature of increased afferent excitability (Figure 3F, variation in ASIS height, week four; Figure 4B, mean ankle angle during swing, week two). The divergence of controls and DREADDs animals in weeks four to six (Figures 3A,F,G, 4D,F) likely reflect the critical transitory phase where plasticity can be more strongly influenced, after which changes are more difficult to induce. During this phase, the nervous system is likely learning to adapt to the increased excitability of the afferents, attempting to make this input functional. After increased variability in ASIS height in week 4 (Figure 3F), ASIS height is stabilized closer to baseline (Figure 3A). For ankle angle, however, increased excitability pushes it further from baseline, to a more flexed angle, before recovery brings it back in line with controls (Figure 4B). The nature of increased excitability with DREADDs comes into play here; whether it tonically activates neurons or leads to more extended bursts of activity after a phasic activation, depending on dose, may influence this transition period and is an interesting point for future study, and for comparison with phase-locked methods of stimulation.

New and strengthened synapses in Clarke's column by hM3Dq DREADDs activation may have implications for relaying sensory information to supraspinal centers during locomotion. Under normal conditions, the dorsal spinalocerebellar (dSC) tract neurons within Clarke's column relay sensory information to cortical motor centers for error adjustments and motor learning. Thus, in the injured condition, plasticity of targeted afferents, including groups Ia and Ib proprioceptors as well as group II cutaneous afferents, could plausibly more efficiently relay information about hindlimb position and movement to dorsal spinalocerebellar (dSC) tract neurons (Kim et al., 1986; Aoyama et al., 1988; Edgley and Gallimore, 1988; Bosco et al., 2000; Bosco and Poppele, 2003; Hantman and Jessell, 2010; Sengul and Watson, 2012). Future studies that record brain activity may help to unearth the effects of increased activation of peripheral afferents on supraspinal centers, particularly those involved in motor learning and error correction. Importantly, upregulation of large diameter peripheral afferents by DREADDs may be working similarly to activation by electrical stimulation. A hallmark of the EES paradigm is its ability to promote plasticity and modulate neural circuitry for use in locomotor tasks (Harkema et al., 2011). Higher order neural centers could filter uniformly boosted afferent input to use these enhanced sensory cues appropriately. Circuitry that is capable of siphoning general hindlimb-mediated afferent upregulation may also reside within lumbar CPGs or other spinal processing centers (Capogrosso et al., 2013). Like EES, afferent activity elevated by DREADDs activation may also be interpreted by neural circuitry appropriately.

In our study, we observed that joint angle variation (here computed as the standard deviation of the joint angle time series for a stride; so a measure of range of motion of the joint angle) within the DREADDs group was more pronounced in angles of the hip joint (Figure 4F) and smaller in the ankle joint (Figures 4C,D). These differences across joints could reflect differences in activation of muscles given the innervation of the DRGs that we transduced; or it could reflect differences in muscle mass about the different joints. Motor noise is multiplicative (Valero-Cuevas et al., 2009); therefore if we had injected equal amounts of additional activation across the motor pools, the larger muscles about the hip could inject more noise than smaller muscles about the ankle, for a given level of activation, resulting in larger ranges of motion; however it may still be expected that both hip and ankle would be larger in DREADDs than controls.

Elimination of large diameter peripheral afferent activation after SCI has been demonstrated to induce significant kinematic changes. For example, sensorimotor improvements reported with EES are enabled when stimulation is turned on and tuned to a specific frequency and amplitude (Harkema et al., 2011; Capogrosso et al., 2013; Angeli et al., 2014; Formento et al., 2018). Additionally, studies with proprioceptive ablation following recovery from SCI permanently reverts improvements in functional recovery to the injured state (Takeoka et al., 2014; Takeoka, 2019; Takeoka and Arber, 2019). Our results corroborate these findings. In our study, we report that withdrawal of DREADDs agonist CNO results in a grossly observed “buckling” or “collapse” event of the hindquarters during treadmill locomotion, a phenomenon reported in mice with spinal cord injury (Basso et al., 2006). We qualitatively define the buckling phenotype as follows: DREADDs animals would take one to several normal strides; suddenly drop their hindquarters onto the treadmill belt and remain seated on the belt moving their hindlimb as if they were mid-stride, but unable to stand; correct their error by lifting their hindquarters off the belt; and begin locomoting normally again (Figure 5). The range of overall hindquarter height (see ASIS height) in the absence of hM3Dq DREADDs activation was larger due to the buckling event. It is interesting to speculate about the source of this phenotype. One simple interpretation is that adaptations have occurred to perform an activity (e.g., locomotion) with a pattern of peripheral afferent activity induced by DREADDs activation. As such, the neural circuitry might be forced to compensate differently in the absence of this pattern of activity. Another interpretation is that the lack of DREADDs activation generates less stiff hindlimb musculature, which in turn results in the collapsing of the hindquarters (Latash, 2018). Furthermore, weight support mechanisms are potentially driving the buckling event. With DREADDs activation, afferent input may be sufficient to avoid buckling events. By extension, in the absence of DREADDs activation, there may be insufficient afferent feedback to prevent loss of weight support (Norton and Mushahwar, 2010; De Leon and Dy, 2017). Finally, the absence of DREADDs activation in cutaneous afferents in the footpad may have implications on shifts the body produces to compensate for the lack of expected tactile information, resulting in the buckling event (Park et al., 2019). Results suggest that DREADDs activation is helpful, but perhaps not required, to access newly formed and strengthened pathways since DREADDs animals demonstrate the ability to lift their hindquarters up off the treadmill belt (e.g., the termination of the buckling event). This may indicate there are underlying circuit changes that are accessible for motor correction even in the absence of DREADDs activation. As such, continued exercise training in the absence of DREADDs activation may eliminate the buckling phenotype altogether. It is entirely possible that the combination of DREADDs activation *and* neural circuit plasticity are required to prevent a buckling event from occurring.

Outside of the buckling phenomenon, we did not observe dramatic changes in function with withdrawal of DREADDs activation in week 7 (Figures 3, 4: compare weeks 6, 7, and 8; including orange and blue shaded weeks). Features in DREADDs animals that had risen above or below controls stayed there (above: Figures 3A–E,G,H, 4F; below: Figures 3F, 4D). This could indicate that underlying plasticity has “taken over” in supporting the changes we observe in kinematics in later weeks, and that DREADDs activation is no longer necessary.

## Conclusion

Sensorimotor improvements after SCI are often marked by plasticity of damaged and spared neural circuitry. In this study, we demonstrate the use of hM3Dq (excitatory) DREADDs in lumbar large diameter peripheral afferents in a SCI model, similar to epidural electrical stimulation of the lumbosacral spinal cord. Unlike electrical stimulation, genetic techniques enable for characterization of neural pathway changes that occur with enhanced afferent feedback, such as our observation of increased plasticity within motor pools and Clarke's column in animals with DREADDs. This plasticity might underlie kinematic differences between DREADDs animals and controls that we observe, such as increased height of the hindquarters as well as more appropriate ankle joint movements during the step cycle. Future studies could further trace the mechanisms of plasticity and utilize muscle recordings to illuminate where DREADDs is most influential in inducing biomechanical changes.

## Data availability statement

The raw data supporting the conclusions of this article will be made available by the authors, without undue reservation.

## Ethics statement

The animal study was reviewed and approved by Temple University Institutional Animal Care and Use Committee.

## Author contributions

JE, ML, GS, and AS contributed to conception and design of the study. JE, HS-B, SS, JC, TC, RS, GM, BR, and AS performed surgeries and experiments, collected data, and contributed to figure generation. JE and AS performed the statistical analysis and wrote sections of the manuscript. JE, RS, ML, GM, and AS contributed to revision of the manuscript. All authors read and approved the submitted version.

## Funding

This work was supported by NIH NINDS grant 1R01NS114007-01A1 to AS, NIH NINDS grant R01NS117749-01 to GS, and NIH/NINDS grant NS114007 to ML, Shriners Hospitals for Children Grant #85115, Craig H. Neilsen Foundation Senior Research Grant (#546798) to AS, Craig H. Neilsen Foundation Project #598563 to ML, and the Shriners Viral Core Grant #84051-PHI-21 to GS.

## Conflict of interest

The authors declare that the research was conducted in the absence of any commercial or financial relationships that could be construed as a potential conflict of interest.

## Publisher's note

All claims expressed in this article are solely those of the authors and do not necessarily represent those of their affiliated organizations, or those of the publisher, the editors and the reviewers. Any product that may be evaluated in this article, or claim that may be made by its manufacturer, is not guaranteed or endorsed by the publisher.

## Supplementary material

Supplementary Figure S1 Identification of virally transduced DRG neurons by colabeling with CGRP **(A)** and Parvalbumin **(C)**, and a thermal nociception assay **(B). (A)** Immunohistochemical (IHC) colabeling of DRG neurons with CGRP **(A, left)**, neurons transduced by our viral construct (AAV2-hSyn-hM3Dq-mCherry; **(A, middle)**, and merge **(A, right)**. We quantified the proportion of mCherry positive cell bodies that were also CGRP positive, finding this to 6.6 ± 4.9% of cells (mean ± SD; N = 10 DRGs from five rats, range one to three DRGs per rat). **(C)** IHC colabeling of DRG neurons for Parvalbumin (PV; **C, left)**, neurons transduced by our viral construct **(C, middle)**, and merge **(C, right)**. The proportion of mCherry positive cell bodies that were also PV positive was 59.1 ± 16.6% of cells (mean ± SD; N = 10 DRGs from five rats, two DRGs per rat). **(B)** To ensure that activation of our virally transduced afferents by DREADDs does not cause a significant increase in nociceptive sensitivity, we carried out a standard Hargreaves thermal nociception assay (adapted from Eisdorfer et al., 2021, with detailed methods described therein; also see Goh et al., 2015). We found that activation of our DREADDs with injection of CNO (4 mg/kg) did not significantly decrease the paw withdrawal time (one way repeated measures ANOVA; p = 0.44; F(2,10) = 0.95; N = 6 rats.

Supplementary Figure S2 cFOS staining in spinal cord sections to confirm excitation of afferents by DREADDs. At present, protein staining for cFOS with IHC in the dorsal root ganglia (DRG) is very difficult in rats. To our knowledge, there are no readily available antibodies that currently work reliably for this stain. As such, to perform additional verification that the DREADDs are exciting afferents in our injected DRGs, we validation activation of afferents with a cFOS stain in the spinal cord segments that are innervated by afferents from our injected DRGs, in additional rats that did not have spinal cord lesions. Having injected into the DRGs from spinal roots L3-L5 in these naïve rats, we stained for cFOS in spinal cord sections at vertebral levels T13-L1, where these roots innervate the spinal cord. Two “experimental” rats **(A,B,E,F)** received excitatory DREADDS injections into these DRGs (AAV2-hSyn-hM3Dq-mCherry), and then three weeks later were injected with CNO (4 mg/kg), then after 30 minutes wait ran on a treadmill for one hour at 16 cm/s, and then after a one hour wait were perfused and the cFOS staining carried out. Two control rats **(C,D,G,H)** consisted of one **(C,G)** having had DRGs injected with an mCherry sham (AAV2-hSyn-mCherry), and another having no DRG injections **(D,H)**. Control rats received an identical protocol before perfusion; they were administered CNO and ran on the treadmill at the same speed and with the same experimental timing. cFOS images were processed in ImageJ; images were converted to grayscale, thresholded, and the particle analyzer with restrictions for size and circularity used to count cFOS+ cells. Experimental sections contained 144 **(A,E)** and 125 **(B,F)** cFOS+ cells, whereas control sections contained 61 **(C,G)** and 88 **(D, H)** cFOS+ cells. A one-sided unpaired t-test for the former two samples being larger than the second two samples is significant, giving t = 3.6 and p = 0.039, and suggesting that our DREADDs activation of afferents is functioning and influences these cells in the spinal cord.

Supplementary Figure S3 Dependence of kinematic parameters on spared spinal cord tissue area. Fits of mean values of kinematic variables (one value per animal) at six weeks post injury and speed 16 cm/s against lesion spared tissue area (mm^2^). Significance and other parameters for these models are listed in Table 1, and overlaid here are the fit line with confidence intervals. None of these approached significance, suggesting the lesion size was not causing significant variation in these kinematic variables. These plots show several of the variables seen in Figures 3, 4, whereas Table 1 lists model parameters for all of them.

Supplementary Figure S4 Dependence of kinematic parameters on transduction efficiency of DRG afferent neurons. Fits of mean values of kinematic variables (one value per animal) at six weeks post injury and speed 16 cm/s against DRG neuron transduction efficiency (percent). Significance and other parameters for these models are listed in Supplementary Table S1, and overlaid here are the fit line with confidence intervals. These plots show several of the variables seen in Figures 3, 4, whereas Supplementary Table S1 lists model parameters for all of them. Here only standard deviation of ankle angle during swing (*SD Ankle Angle Swing)* is statistically significant, while standard deviation of ASIS height (*SD Asis Height)* is close to significant. Note that these results are within excitatory DREADs animals only, and support the conclusion that the DREADDs excitation is causing the observed changes in kinematics seen in Figures 3–5. The variable *SD Ankle Angle Swing* was not significant in the control group (p = 0.73, n = 6), as expected as these animals did not have excitatory DREADDs. The fact that the *SD Ankle Angle Swing* is positively correlated with transduction efficiency suggests that the DREADDs excited animals may be more able to move their ankle through a large range of motion during swing. For *SD Asis Height* which approached significance (p = 0.06), it may be that the level of DREADDs afferent excitation influences how much the height of the pelvis is controlled and or stabilized, and is therefore less variable; however the difference here of one mm is unlikely to be highly biologically relevant.

Supplementary Table S1 Dependence of kinematic parameters on lesion properties and dorsal root ganglion neuron transduction efficiencies. Mean values of kinematic variables (one value per animal) at week six post injury and speed 16 cm/s were fit against lesion spared tissue remaining (mm^2^) and DRG transduction efficiency (percent of neurons) using the R *lm* function. The estimate of the slope (“Estimate”) term of the model for each variable is displayed, with associated metrics of the model fit and significance. None of the models against spared tissue approach significance, suggesting the lesion size was not causing significant variation in these kinematic variables. For DRG transduction efficiency, only standard deviation of ankle angle during swing is statistically significant, while standard deviation of ASIS height is close to significant. Note that these results are within excitatory DREADs animals only, and support the conclusion that the DREADDs excitation is causing the observed changes in kinematics seen in Figures 3–5. This variable (*SD Ankle Angle Swing)* was not significant in the control group (p = 0.73, n = 6), as expected as these animals did not have excitatory DREADDs.
