# Supplementary material for: Chemogenetic modulation of sensory afferents induces locomotor changes and plasticity after spinal cord injury
Source: Front Mol Neurosci. 2022 Aug 26;15:872634. doi: 10.3389/fnmol.2022.872634 (PMC9461563; doi:10.3389/fnmol.2022.872634)
Supplement: Supplementary Table S1 — Dependence of kinematic parameters on lesion properties and dorsal root ganglion neuron transduction efficiencies. Mean values of kinematic variables (one value per animal) at week six post injury and speed 16 cm/s were fit against lesion spared tissue remaining (mm2) and DRG transduction efficiency (percent of neurons) using the R lm function. The estimate of the slope (“Estimate”) term of the model for each variable is displayed, with associated metrics of the model fit and significance. None of the models against spared tissue approach significance, suggesting the lesion size was not causing significant variation in these kinematic variables. For DRG transduction efficiency, only standard deviation of ankle angle during swing is statistically significant, while standard deviation of ASIS height is close to significant. Note that these results are within excitatory DREADs animals only, and support the conclusion that the DREADDs excitation is causing the observed changes in kinematics seen in Figures 3–5. This variable (SD Ankle Angle Swing) was not significant in the control group (p = 0.73, n = 6), as expected as these animals did not have excitatory DREADDs. [file Table_1.pdf]

Kinematic variables versus spared tissue and transduction efficiency

Week 6, Speed 16 cm/s

	Spared Tissue					Transduction Efficiency				
Variable	Estimate	Std. Error	P Value	R^2	N	Estimate	Std. Error	P Value	R^2	N
Max Asis Height	−2.88	4.22	0.51	0.04	[1] 13	−80.35	115.88	0.54	0.14	[2] 5
Mean Ankle Swing	−6.86	5.58	0.24	0.11	14	192.37	246.06	0.49	0.17	5
Mean Asis Height	−2.69	4.62	0.57	0.03	13	−73.81	102.74	0.52	0.15	5
Mean Asis Height Stance	−3.22	4.74	0.51	0.04	13	−89.60	102.84	0.45	0.20	5
Mean Asis Height Swing	−1.38	4.27	0.75	0.01	13	−34.17	96.09	0.75	0.04	5
Mean Hip Height Swing	−3.48	3.81	0.38	0.08	12	−17.02	89.58	0.86	0.01	5
Mean Knee Height	−2.56	3.26	0.45	0.05	14	−68.96	68.79	0.39	0.25	5
Min Asis Height Stance	−1.99	4.76	0.68	0.02	13	−110.64	96.58	0.34	0.30	5
Step Duration	−0.02	0.05	0.74	0.01	13	0.01	0.73	0.99	0.00	4
SD Ankle Angle	−0.52	1.01	0.61	0.02	14	13.88	22.45	0.58	0.11	5
SD Ankle Angle Swing	−3.83	4.21	0.38	0.07	13	169.23	46.81	0.04	0.81	5
SD Asis Height	−0.09	0.40	0.83	0.00	13	−18.64	4.63	0.06	0.89	4
SD Hip Angle	−0.52	1.01	0.61	0.02	14	13.88	22.45	0.58	0.11	5
SD Knee Angle	−0.83	1.25	0.52	0.04	13	−40.80	41.95	0.40	0.24	5

[1] Control and Excitatory DREADDs animals

[2] Excitatory DREADDs animals only
